# A Quick Look Back at the Microalgal Biofuel Patents: Rise and Fall

**DOI:** 10.3389/fbioe.2020.01035

**Published:** 2020-08-26

**Authors:** Dongzi Li, Wei Du, Wantao Fu, Xupeng Cao

**Affiliations:** ^1^Marine College of Science-Technology and Environment, Dalian Ocean University, Dalian, China; ^2^Dalian Institute of Chemical Physics, Chinese Academy of Sciences, Dalian, China; ^3^State Key Laboratory of Catalysis, Dalian Institute of Chemical Physics, Chinese Academy of Sciences, Dalian, China; ^4^Dalian National Laboratory of Clean Energy, Division of Solar Energy, Dalian Institute of Chemical Physics, Chinese Academy of Sciences, Dalian, China; ^5^Dalian Key Laboratory of Energy Biotechnology, Dalian Institute of Chemical Physics, Chinese Academy of Sciences, Dalian, China

**Keywords:** microalgae, patent, industrialization, stress cultivation, biofuel

## Abstract

Microalgae is a promising organism as the feedstock of the next generation biofuels, as well as high value nature products, such as astaxanthin, normally under certain stress cultivation conditions. With the clear industrialization targets, there have been two waves of microalgae R&D from the last century and showed obvious energy-driven trends. The overall R&D came into a valley now, however, the promising is still there. So here, from the industrialization point of view, the patent evolution concerning the microalgae for biofuels in the second wave were reviewed and summarized. These technology information will help the scientists to join together with industry to drive the next boost.

## Introduction

Microalgae are found in both marine and freshwater environments and convert carbon dioxide to potential biofuels (carbohydrates, lipids), foods and high-value products, driven by solar energy, as well as are good organisms for bioremediation applications (Sheehan et al., 1998; Chisti, 2007; Hu et al., 2008; Mata et al., 2010; Wijffels and Barbosa, 2010; Chen et al., 2018; Khan et al., 2018; Shuba and Kifle, 2018). With the afraid of fossil fuel crisis, it’s officially considered as a solution at 1978 under the program funded by the US Department of Energy’s Office of Fuels Development, known as the Aquatic Species Program (or ASP), which main focus was the production of biodiesel from high lipid-content algae grown in ponds, utilizing waste CO_2_ from coal fire power plants (Sheehan et al., 1998). Over the nearly two decades (from 1978 to 1996), ASP stopped far from the industrialization of microalgae as fuels stocks, one reason for which was the high life-span cost comparing with the relative low price of the fuels. However, ASP promoted the R&D of microalgae from the collection and evaluation of thousand species, the science of manipulating the genes and metabolism, to the engineering of production systems. The detailed contributions of ASP was well-documented Sheehan et al. (1998).

While the oil price dramatically increased from the beginning of the twenty-first century, microalgae come back the visual field again with the support from governments, the industries and the venture capitals all over the world, but cooled down after 2015 till now, indicated by the takeover of Solazyme by TerraVia in US and retreat of ENN in China. The rise and fall of it was the result of multi factors, which was not only the affair of science and technology. As one of the key components of the innovation, the patent bridges the lab and the factory. Here, after the second wave of microalgae R&D, the patent evolution during the last 20 years were quickly reviewed for the reference to the next boost of microalgae industrialization.

## The Analysis of Microalgae Biofuels Related Patents From Last 20 Years

### Patents Collection and Refinement

The patents data used here were mainly from Patsnap^®^ patent database and Derwent World Patents Index was used as an amendment. The keywords used for index were provided in Supplementary Data File. More than ten thousand patents were further refined, considering the fact that microalgae biofuels production is the process to convert solar energy to chemical energy by photosynthesis in a direct or indirect manner, the photosynthesis related information should be shown in the claims, such as autotrophic growth, or with light, etc.

Differing to normal research publications, the patent is a kind of legal documents and a collection of patent applications covering the same or similar technical content were defined as a family, which can help us to focus on the technical part of patents, other than the different legal status. After refine, 3,393 items from 730 families were extracted for the further analysis. To verify the coverage of the index, 11 patents from authors were used as a reference, which covers the strain screening and application to the downstream bio-refinery to biofuels, and all of them were included by above index.

### The Patent Application Amount Change With Crude Oil Price

The aim of the development of biofuels is the makeup or replacement of traditional fossil fuels, especially the oil, for the reason of the final products of the biofuels. So the difference between the cost of biofuels to oil should be a driving force of the R&D development of microalgae.

**FIGURE 1:**
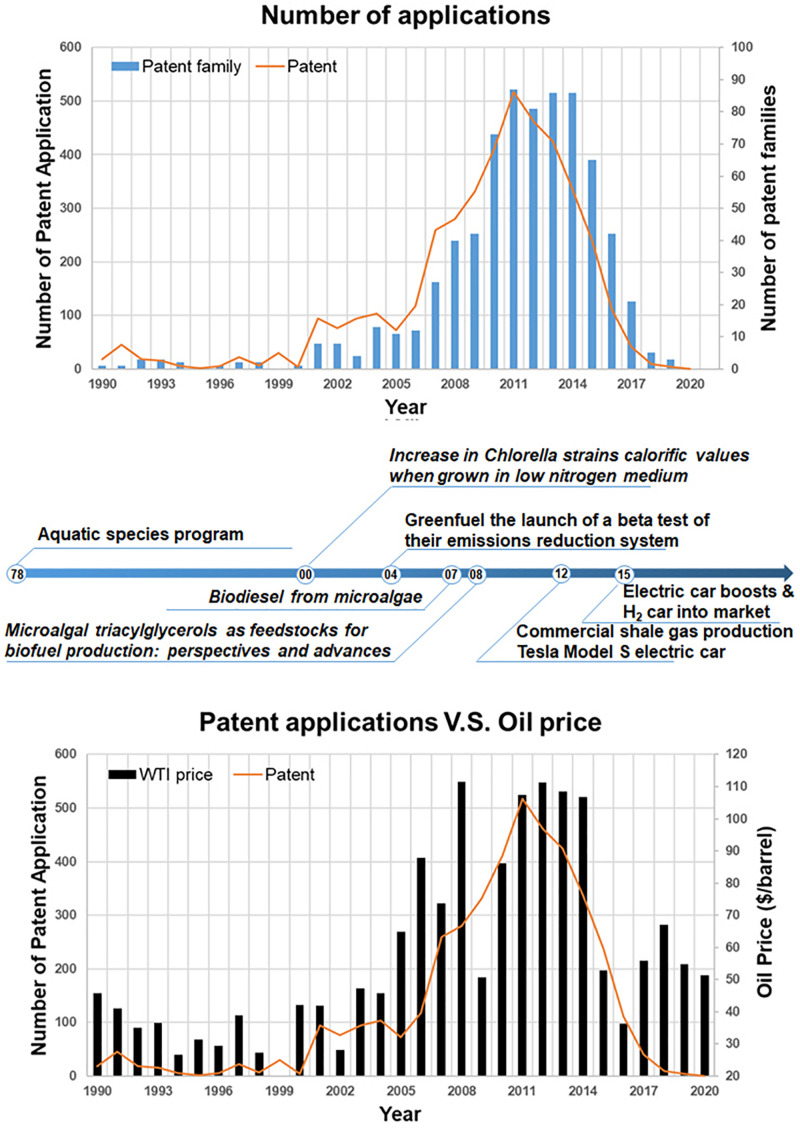
The patent application changes in last 30 years. The upper figure is the application in terms of single patent and patent family. The bottom figure is the application changes with the crude oil prices. The timeline in the middle marks some milestones for microalgae biofuel R&D development.

The crude oil prices per barrel of West Texas Intermediate (WTI) with the adjustment for inflation using the headline CPI were collected from Macrotrends database^1^. For simplification, the average price of January of each were used to compared with the yearly patent application status. In Figure 1, the overall correlation of oil price to the number of patent application is about 0.86 before 2015 and 0.84 till now.

Although only a little changes in the correlation factors shown in the last 5 years, serious effects on the direction of microalgae R&D existed. It will detailed discussed in the “Discussion” section.

### Global and Technical Distributions

Nearly 25% patents were held by United States (975) followed by Japan (541), China (289), Australia (164), Canada (155), and South Korea (119). The validated patent number was shown in Supplementary Figure 1, briefly, more than half of them are validated till the end of June, 2020.

As an intellectual property (IP) tools, it’s reasonable that most top player of patent applications were companies, such as Solazyme Inc. (now is acquired by Corbion) with 313 items and nearly 10% of the patent pool analyzed (see Supplementary Table 1). However, the Scripps Research Institute also holds 46 items and ranked first among research institutes.

To obtain an overall technical view of these patent, International Patent Classification (IPC^2^) information was used to classify and summarize the patents in the pool. The top related IPC classes were listed in Supplementary Table 2 and the IPC class distribution in different countries or organizations were shown in Supplementary Figure 1. The yearly distribution of major IPC was shown in Supplementary Figure 2. Briefly, the majority of patents are related to strains and their cultivation under different condition, especially some stress conditions, the introduction of some regulating reagents, the combination of different downstream treatment and bio-refinery methods or processes.

The most related class is C12N, which is directly related to microalgae itself, including compositions, propagating, preserving, or maintaining, and also including mutation or genetic engineering of microalgae as well as novel culture media. In the patent pool, 58% patents were concerned with this class. The second hot class is C12P, which relates to the processes to synthesis a desired chemical compound or composition or to separate optical isomers from a racemic mixture. It consisted with the aim of these patent to produce biofuels, which are mostly in the form of fatty acids or lipids, or carbohydrates. Among them, some multifunctional compounds, other than the feedstock to biofuels, such as special carbohydrates, pigments or medium chain fatty acids, were drawing more attentions.

After more than thousands of strains, there was no natural microalga suitable for the biofuel production directly. So in the last two decades, the artificial modification of natural strains was also concerned, especially with the development of gene engineering, such as CRISPR technology in US20160208243A1 family. So the IPC A01H reached the third rank in the second wave.

### Core Techniques Within Patents for Microalgae Biofuels Based on Citation Relationships

The citation of the patent usually represented the core techniques in the field and direction of the knowledge flow (Alcácer and Gittelman, 2006). The citation in the patent is not as strict as scientific publications, however, the core techniques cannot be ignored due to their potential legal risks. In the patent pool, the citation relationship were summarized by using Cytoscape (version 3.8.0). Some important knots were significantly shown with concentrated links (Supplementary Figure 3).

The most cited validated patents, with cited number over 100, were listed in the Table 1. Among them, only US20160208243A1 was applied in 2015, and other are almost older than 10 years and close to the edge of patent term. In this 16 top cited patents, five of them were about the gene manipulation of microalgae, eight of them were about the cultivation to promote the biofuel production and three of them were concerned with the downstream bio-refinery. Bio-refinery has the advantages of high atomic economy and was expected as an integrated solution for energy, wastewater treatment, food and nutrient supply (Suganya et al., 2016). However, maybe limited by the biomass supply, except on the health-related approaches, there was few reports on the stable industrialization of bio-refinery or co-production established.

**TABLE 1:** Valid patents with more than 100 cites.

No.	Patent number	Year	Cited number	Title	Patent Owner	Description
1	US6750048	2001	361	Solventless extraction process	OMEGA TECHNOLOGIES	A method for extracting lipids from microorganisms without using organic extraction solvent.
2	US20080160593A1	2007	354	Two-stage process for producing oil from microalgae	GENIFUEL CORPORATION	By promoting sequential photoautotrophic and heterotrophic growth, a biofuels production process from cultivating an oil-producing algae.
3	US20040019927A1	2003	276	Polynucleotides and polypeptides in plants	MENDEL BIOTECHNOLOGY, INC.	About plant transcription factors and using to produce transgenic plants with advantageous properties.
4	US20090148918A1	2008	220	Glycerol feedstock utilization for oil-based fuel manufacturing	CORBION BIOTECH, INC.	Methods of manufacturing biodiesel and other oil-based compounds using glycerol and its combinations as an energy source in fermentation of oil-bearing microorganisms.
5	US20050009049A1	2004	216	Expanding the eukaryotic genetic code	SCRIPPS RESEARCH INSTITUTE	Compositions and methods for producing translational components that expand the number of genetically encoded amino acids in eukaryotic cells.
6	US20080160591A1	2007	204	Diffuse light extended surface area water-supported photobioreactor	COLORADO STATE UNIVERSITY RESEARCH FOUNDATION	A scalable photobioreactor system for efficient production of photosynthetic microorganisms such as microalgae and cyanobacteria.
7	US7883882	2009	196	Renewable chemical production from novel fatty acid feedstocks	CORBION BIOTECH, INC.	Methods of manufacturing renewable chemicals through the manufacture of novel triglyceride oils followed by chemical modification of the oils.
8	US20090047721A1	2008	186	Renewable diesel and jet fuel from microbial sources	CORBION BIOTECH, INC.	Methods of manufacturing alkanes from triglyceride oils produced through fermentation of oil-bearing microbes.
9	US20090011480A1	2009	185	Use of cellulosic materials for cultivation of microorganisms	CORBION BIOTECH, INC.	Methods of cultivating oil-bearing microbes using cellulosic material.
10	US20040265952A1	2004	163	Unnatural reactive amino acid genetic code additions	ENERGY, UNITED STATES DEPARTMENT OF	Compositions and methods for producing translational components that expand the number of genetically encoded amino acids in eukaryotic cells.
11	US20160208243A1	2015	162	Novel CRISPR enzymes and systems	THE BROAD INSTITUTE INC.; PRESIDENT AND FELLOWS OF HARVARD COLLEGE; MASSACHUSETTS INSTITUTE OF TECHNOLOGY	Systems, methods, and compositions for targeting nucleic acids, including microalgae.
12	US20110092726A1	2011	137	System for cultivation and processing of microorganisms, processing of products therefrom, and processing in drillhole reactors	WINWICK BUSINESS SOLUTIONS PTY LTD	Cultivating autotrophic microorganisms, particularly microalgae, in a bioreactor to enable laminar flow which in cross section is closed and which has transparent walls through which the culture is irradiated to enable photosynthesis.
13	US20030037355A1	2001	131	Methods and compositions to modulate expression in plants	SCRIPPS RESEARCH INSTITUTE; SYNGENTA PARTICIPATIONS AG	The use of zinc finger proteins and fusions of said proteins to regulate gene expression and metabolic pathways.
14	US20110086386A1	2010	124	Algae biomass fractionation	TRUCENT, INC.	A method of fractionating biomass, and recovering cell and cell derived products from the non-polar solvent solution and polar biomass solution.
15	US20080118964A1	2006	117	Continuous-batch hybrid process for production of oil and other useful products from photosynthetic microbes	CELLANA, INC.	Cultivating photosynthetic microbes in closed systems for continuous cultivation and open systems for batch cultivation.
16	US20100151538A1	2009	107	Cellulosic cultivation of oleaginous microorganisms	CORBION BIOTECH, INC.	Cultivation of oleaginous microorganisms on feedstocks including depolymerized cellulosic material such as corn stover, Miscanthus, forage sorghum, sugar beet pulp, and sugar cane bagasse.

## Discussion

### The Driving Force for the Future Microalgae R&D Changes

As shown in Figure 1, at the beginning, the afraid of depletion of fossil fuels drove the first wave of microalgae R&D, mostly under the support of ASP by the US DOE and more likely a pure scientific affair to produce bio hydrogen or bio transportation fuels. The higher cost of microalgae cultivation and relative low oil price caused the cease of the first wave in 1995 (Sheehan et al., 1998). But, the accumulation on strains and numerous technologies helps the form of the second wave while the crude oil price dramatically increased in the early of 21 century.

The stress condition cultivation became the sign of the second wave, mostly started by Illman et al.’s (2000) work on the low nitrogen cultivation for high calorific value biomass to develop renewable energy resource. Differed to the first wave, with the increase of oil price, the microalgae biofuel R&D became a move by both scientists and venture capitals (VCs), which was marked by the launch of the beta test of CO_2_ and NO_x_ emission reduction system by Greenfuel in MIT in 2004. Following Chisti and Hu et al.’s direction in 2007–08 (Chisti, 2007; Hu et al., 2008), around the stressed cultivation of microalgae, a large amount patents were applied and covered the whole processes of microalgae biofuel production. The driving force of this period was still the high crude oil price.

Things changed from 2012, when commercial shale gas realized and Tesla Model S electric car came into the market, which caused the energy production and consuming structure reformed. With less progress in the cut of production cost or significant increase in production efficiency, the VCs and big companies turned their focus. Without enough support, the patent application decrease rapidly.

After 2015, although the oil price increased a little bit, the popularization of electric car and the development of other renewable energy resources caused the traditional driving force of microalgae R&D lost.

### New Driving Force From Synthetic Biology and Customized Natural Products Production

The development of biotechnology brings the hope to meet the next wave of microalgae R&D. Due to its fast growth, unicellular form, high photosynthesis capacity and diverse secondary metabolisms, the microalgae based CO_2_ and NO_x_ capture will be a new driving force for the innovation of a new gene manipulation, cell or organelle factory building, non-meat protein production, medium length chain fatty acids synthesis. And above targets also consisted with the major technical classes, including C12N15, C12P7, and C12N1.

## Author Contributions

DL and XC collected and manually classified the patents and drafted the manuscript. WD organized and guided the analysis. WF analyzed the collected patents and drafted the manuscript. All authors contributed to the article and approved the submitted version.

## Conflict of Interest

The authors declare that the research was conducted in the absence of any commercial or financial relationships that could be construed as a potential conflict of interest.

## Footnotes

1 https://www.macrotrends.net/1369/crude-oil-price-history-chart

2 https://www.wipo.int/classifications/ipc/en/
