# Supplementary material for: A Quick Look Back at the Microalgal Biofuel Patents: Rise and Fall
Source: Front Bioeng Biotechnol. 2020 Aug 26;8:1035. doi: 10.3389/fbioe.2020.01035 (PMC7479055; doi:10.3389/fbioe.2020.01035)
Supplement: Supplementary file 1 [file Data_Sheet_1.docx]

Supplementary Material

# Supplementary Data

## The keywords for patent collection in Patsnap^®^ database

**First step search:**

( (IPC:(C10G3/00 ) OR ABST:((production OR extraction) AND (diesel OR lipid OR oil)) OR TTL:((production OR extraction) AND (diesel OR lipid OR oil)) OR CLMS:((production OR extraction) AND (diesel OR lipid))) AND (ABST:((oil $ws microorgani*) OR microalg* OR (engineered $ws microalg*)) OR TTL:((oil $WS microorgani*) OR microalg* OR (engineered $WS microalg*)) OR CLMS:(microorgani* OR microalg* OR engineered $ws microalg*) OR IPC:(C12n1)) AND PBD:[19800101 TO 20200630])

Results: **13,963** items and **6,090** families

**Second step search:**

(( (IPC:(C10G3/00 ) OR ABST:((production OR extraction) AND (diesel OR lipid OR oil)) OR TTL:((production OR extraction) AND (diesel OR lipid OR oil)) OR CLMS:((production OR extraction) AND (diesel OR lipid))) AND (ABST:((oil $ws microorgani*) OR microalg* OR (engineered $ws microalg*)) OR TTL:((oil $WS microorgani*) OR microalg* OR (engineered $WS microalg*)) OR CLMS:(microorgani* OR microalg* OR engineered $ws microalg*) OR IPC:(C12n1)) AND PBD:[19800101 TO 20200630]) ) AND (photosynthesis)

Results: **1,408** items and **769** families

According to above patents, a pool of 3,393 items from 730 families were built by manual read, classification and refine.

# Supplementary Figures and Tables

**STable 1 The top applicants of microalgae biofuel related patents**

| **Applicant for patent** | **Number of patent** |
| --- | --- |
| SOLAZYME INC* | 313 |
| BASF PLANT SCIENCE GMBH | 98 |
| SAPPHIRE ENERGY INC | 65 |
| FERMENTALG | 64 |
| COMMONWEALTH SCIENTIFIC AND INDUSTRIAL RESEARCH ORGANISATION | 57 |
| THE SCRIPPS RESEARCH INSTITUTE | 46 |
| PRONUTRIA INC | 41 |
| TOYOTA JIDOSHA KABUSHIKI KAISHA | 37 |
| SYNTHETIC GENOMICS INC | 36 |
| BIO ARCHITECTURE LAB INC | 34 |

* Solazyme Inc was finally acquired by Corbion from TerraVia, and the number is the sum of all of them and ignores the complex IP right transfer.

**STable 2 The top IPC classes related**

| **Class** | **Definition** |
| --- | --- |
| A01H1 | Processes for modifying genotypes (A01H 4/00 takes precedence) [2006.01] |
| A01H5 | Angiosperms, i.e. flowering plants, characterised by their plant parts; Angiosperms characterised otherwise than by their botanic taxonomy [2018.01] |
| A21D10 | Batters, dough or mixtures before baking [2006.01] |
| A21D2 | Treatment of flour or dough by adding materials thereto before or during baking (batters, dough or mixtures before baking A21D 10/00) [2006.01] |
| A23B5 | Preservation of eggs or egg products [2006.01] |
| A23D7 | Edible oil or fat compositions containing an aqueous phase, e.g. margarines [2006.01] |
| A23D9 | Other edible oils or fats, e.g. shortenings, cooking oils [2006.01] |
| A23J3 | Working-up of proteins for foodstuffs [2006.01] |
| A23L1 | FOODS, FOODSTUFFS, OR NON-ALCOHOLIC BEVERAGES, NOT COVERED BY SUBCLASSES A21D OR A23B-A23J; THEIR PREPARATION OR TREATMENT, e.g. COOKING, MODIFICATION OF NUTRITIVE QUALITIES, PHYSICAL TREATMENT (shaping or working, not fully covered by this subclass, A23P); PRESERVATION OF FOODS OR FOODSTUFFS, IN GENERAL (preservation of flour or dough for baking A21D) [2006.01] |
| A23L2 | Non-alcoholic beverages; Dry compositions or concentrates therefor; Their preparation (soup concentrates A23L 23/10; preparation of non-alcoholic beverages by removal of alcohol C12H 3/00) [2006.01] |
| A61K31 | Medicinal preparations containing organic active ingredients [2006.01] |
| B01J20 | Solid sorbent compositions or filter aid compositions; Sorbents for chromatography; Processes for preparing, regenerating or reactivating thereof [2006.01] |
| C02F9 | Multistep treatment of water, waste water or sewage [2006.01] |
| C07K14 | Peptides having more than 20 amino acids; Gastrins; Somatostatins; Melanotropins; Derivatives thereof [2006.01] |
| C08L23 | Compositions of homopolymers or copolymers of unsaturated aliphatic hydrocarbons having only one carbon-to-carbon double bond; Compositions of derivatives of such polymers [2006.01] |
| C09K8 | Compositions for drilling of boreholes or wells; Compositions for treating boreholes or wells, e.g. for completion or for remedial operations [2006.01] |
| C10G3 | Production of liquid hydrocarbon mixtures from oxygen-containing organic materials, e.g. fatty oils, fatty acids (production from non-melting solid oxygen-containing carbonaceous materials C10G 1/00) [2006.01] |
| C10L1 | Liquid carbonaceous fuels [2006.01] |
| C11B1 | Production of fats or fatty oils from raw materials [2006.01] |
| C11B3 | Refining fats or fatty oils [2006.01] |
| C11B5 | Preserving by using additives, e.g. anti-oxidants [2006.01] |
| C11D3 | Other compounding ingredients of detergent compositions covered in group C11D 1/00 [2006.01] |
| C12M1 | Apparatus for enzymology or microbiology [2006.01] |
| C12N1 | Microorganisms, e.g. protozoa; Compositions thereof (medicinal preparations containing material from protozoa, bacteria or viruses A61K 35/66, from algae A61K 36/02, from fungi A61K 36/06; preparing medicinal bacterial antigen or antibody compositions, e.g. bacterial vaccines, A61K 39/00); Processes of propagating, maintaining or preserving microorganisms or compositions thereof; Processes of preparing or isolating a composition containing a microorganism; Culture media therefor [2006.01] |
| C12N15 | Mutation or genetic engineering; DNA or RNA concerning genetic engineering, vectors, e.g. plasmids, or their isolation, preparation or purification; Use of hosts therefor (mutants or genetically engineered microorganisms C12N 1/00, C12N 5/00, C12N 7/00; new plants A01H; plant reproduction by tissue culture techniques A01H 4/00; new animals A01K 67/00; use of medicinal preparations containing genetic material which is inserted into cells of the living body to treat genetic diseases, gene therapy A61K 48/00; peptides in general C07K) [2006.01] |
| C12N9 | Enzymes, e.g. ligases (6.); Proenzymes; Compositions thereof (preparations containing enzymes for cleaning teeth A61K 8/66, A61Q 11/00; medicinal preparations containing enzymes or proenzymes A61K 38/43; enzyme containing detergent compositions C11D); Processes for preparing, activating, inhibiting, separating, or purifying enzymes [2006.01] |
| C12P21 | Preparation of peptides or proteins (single-cell protein C12N 1/00) [2006.01] |
| C12P23 | Preparation of compounds containing a cyclohexene ring having an unsaturated side chain containing at least ten carbon atoms bound by conjugated double bonds, e.g. carotenes (containing hetero-rings C12P 17/00) [2006.01] |
| C12P3 | Preparation of elements or inorganic compounds except carbon dioxide [2006.01] |
| C12P39 | Processes involving microorganisms of different genera in the same process, simultaneously [2006.01] |
| C12P5 | Preparation of hydrocarbons [2006.01] |
| C12P7 | Preparation of oxygen-containing organic compounds [2006.01] |
| C12Q1 | Measuring or testing processes involving enzymes, nucleic acids or microorganisms (measuring or testing apparatus with condition measuring or sensing means, e.g. colony counters, C12M 1/34); Compositions therefor; Processes of preparing such compositions [2006.01] |
| G01N21 | Investigating or analysing materials by the use of optical means, i.e. using infra-red, visible or ultra-violet light (G01N 3/00-G01N 19/00 take precedence) [2006.01] |

## Supplementary Figures

##
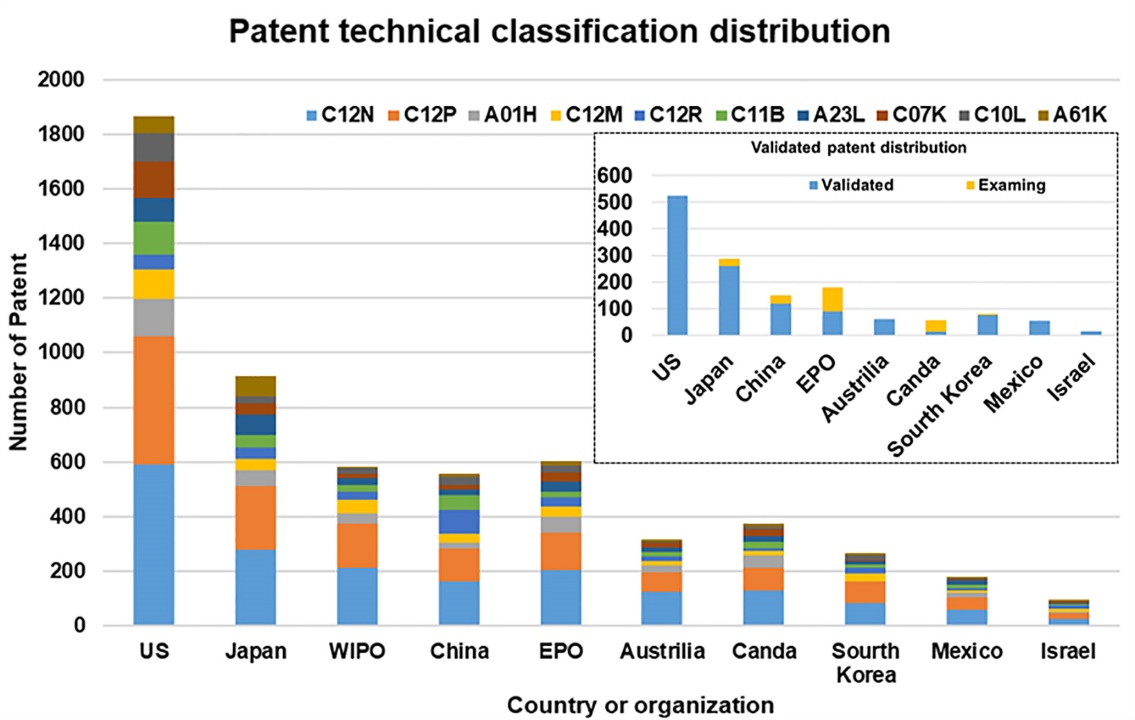


**Supplementary Figure 1.** The technical classification distribution in major countries or organization. The classes shown here were ten major classes in the patent pool. The small figure nearby the right-top corner is the corresponding number of validated patent.


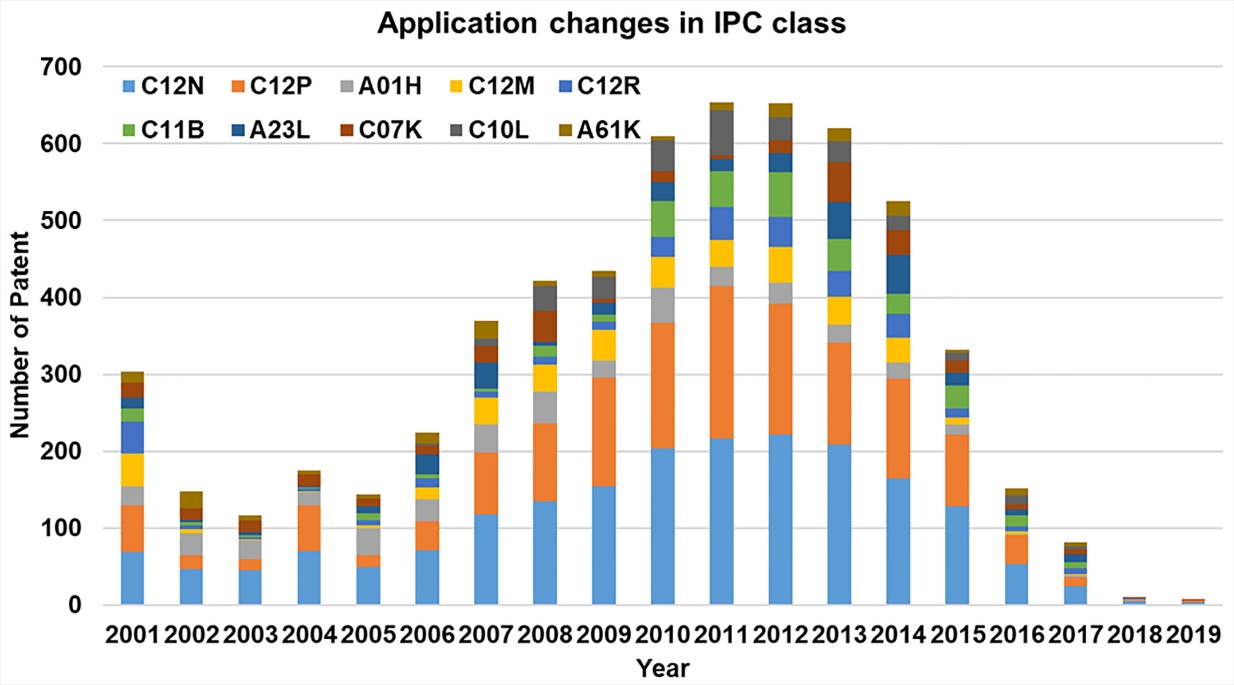


**Supplementary Figure 2.** Application number changes in IPC class with the year. The number and ratio of different technical class indicates the focus shift of microalgae biofuels R&D.


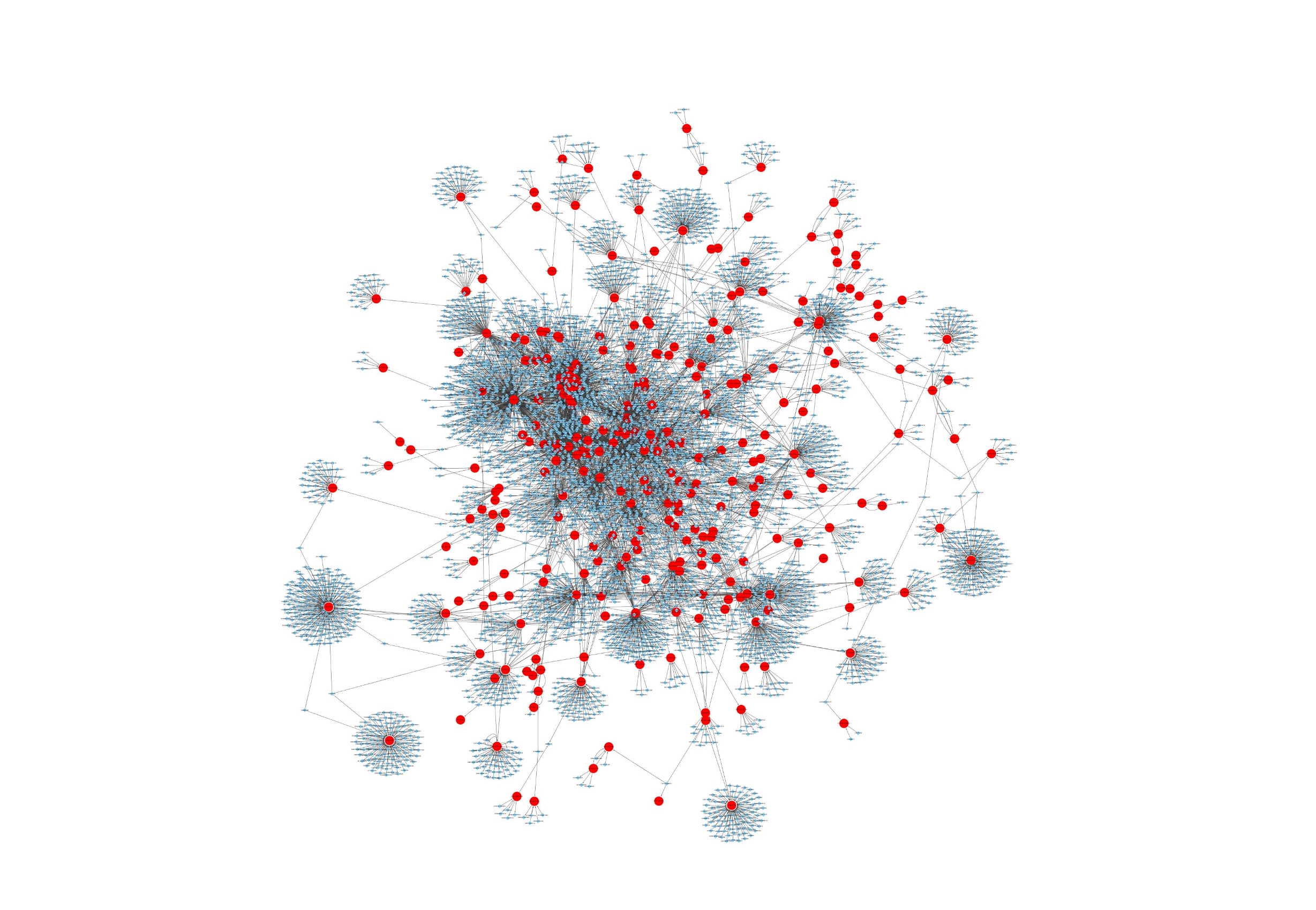


**Supplementary Figure 3.** The major citation network for microalgae biofuel patents. The line between dots indicates the citation relationship between patents and the dots indicates the patents. The most connected knots were selected for further evaluation and listed in Table 1.
